# Allelic differences within DNA polymerase delta subunit 1 correlate with geminivirus resistance in diverse plants

**DOI:** 10.1093/g3journal/jkaf216

**Published:** 2025-09-15

**Authors:** Kerrigan B Gilbert, Patricia Gallardo, Stefanie F King, Cheyenne M Morris, Gabriela L Hernandez, James C Carrington, Rebecca S Bart

**Affiliations:** Donald Danforth Plant Science Center, Saint Louis, MO 63132, United States; Donald Danforth Plant Science Center, Saint Louis, MO 63132, United States; Division of Biology and Biomedical Sciences, Washington University in St. Louis, St. Louis, MO 63130, United States; Donald Danforth Plant Science Center, Saint Louis, MO 63132, United States; Division of Biology and Biomedical Sciences, Washington University in St. Louis, St. Louis, MO 63130, United States; Donald Danforth Plant Science Center, Saint Louis, MO 63132, United States; Division of Biology and Biomedical Sciences, Washington University in St. Louis, St. Louis, MO 63130, United States; Donald Danforth Plant Science Center, Saint Louis, MO 63132, United States; Donald Danforth Plant Science Center, Saint Louis, MO 63132, United States; Donald Danforth Plant Science Center, Saint Louis, MO 63132, United States; UC Berkeley, Berkeley, CA 94720, United States

**Keywords:** virus resistance, allele mining, QTL, geminivirus, DNA polymerase delta 1, Plant Genetics and Genomics

## Abstract

Identifying loci conferring resistance to geminiviruses is an on-going priority in diverse crop species. Multiple geminivirus resistance quantitative trait loci (QTLs) and genes have been described, including most recently DNA polymerase delta subunit 1 (*POLD1*) in both cassava and tomato. From this, we hypothesized that POLD1-mediated resistance is present in a broad range of plant species. An analysis of multiple species with published geminivirus resistance QTLs was done to identify species with POLD1 variation consistent with known resistance alleles. Further, allele mining of over 7,000 accessions across 10 different genera, from both dicots and monocots, identified additional substitutions in key regions of the POLD1 protein as possible novel resistance alleles. These results provide evidence that *POLD1* variation is a source for geminivirus resistance in diverse plants including cotton, soybean, squash, wheat, and maize.

## Introduction

Wild relatives of crop species are a common source of novel resistance traits that can be introduced into breeding programs (García-Arenal and Zerbini 2019). However, viral pathogens often overcome resistance through recombination during mixed infections and genomic mutation (Farooq et al. 2021; Fiallo-Olivé and Navas-Castillo 2023). Resistance that targets an action or sequence-specific domain that is linked to pathogen fitness may be less likely for a pathogen to overcome. An additional consideration is that the ecological context surrounding a resistance trait may impact its durability (Brown 2015; Karasov et al. 2020). Continuing to identify and characterize new sources of resistance will enable breeders to stack resistance genes in elite varieties and may also increase durability.

Crop yields worldwide are negatively impacted by diseases caused by species of the virus family Geminiviridae. Several geminiviruses resistance traits have been reported from diverse plant species (Beam and Ascencio-Ibáñez 2020), and in some cases, the same gene has been identified in multiple species. Examples of geminivirus resistance genes include alleles of a γ-class RNA-dependent RNA polymerase in tomato against tomato yellow leaf curl virus (TYLCV) (Verlaan et al. 2013), in *Capsicum annuum* against pepper yellow leaf curl Indonesia virus and pepper yellow leaf curl Aceh virus (Koeda et al. 2022), and in cucumber against tomato leaf curl New Delhi virus (ToLCNDV) (Koeda et al. 2024). Similarly, mutant versions of the RNA surveillance factor *Pelota* have been characterized as resistance-conferring alleles in both tomato (Ren et al. 2022) and *Ca. annuum* (Koeda et al. 2021), while mutations in *DNA primase large subunit* in multiple species of *Cucumis melo* are linked to resistance to ToLCNDV (Siskos et al. 2023; Pérez-Moro et al. 2024). A nucleotide-binding leucine-rich repeat protein in both tomato and black gram, *Vigna mungo*, confers resistance to TYLCV and Mung bean yellow mosaic virus (MYMV), respectively (Maiti et al. 2012; Yamaguchi et al. 2018). The *PvVTT1* locus, in *Phaseolus vulgaris*, which encodes a protein with a double Toll/interleukin-1 receptor motif, provides dominant resistance to Bean dwarf mosaic virus (Seo et al. 2007).

Recently, a new source of geminivirus resistance was described. In cassava, specific mutations in *DNA polymerase delta subunit 1* (POLD1) were observed to cosegregate with resistance to Cassava mosaic geminivirus, with at least 5 independent, putative resistance alleles reported (Lim et al. 2022). Similar mutations in tomato *POLD1* were recently reported and linked to resistance to the cognate geminivirus pathogen (Shen et al. 2025). *POLD1* is a relatively recently identified susceptibility gene for geminivirus resistance (Wu et al. 2021), and while the exact mechanism of resistance is not known, similar mutations have been studied in yeast and lead to decreased replication rate and/or fidelity (Daee et al. 2010).

Given the large number of plants susceptible to geminiviruses, we speculated that the POLD1-based resistance mechanism may occur outside of cassava and tomato. A computational approach was adopted to investigate this possibility. Here, we report that *POLD1* is present in previously reported QTLs for geminivirus resistance in *Cucurbita moschata* (squash) and *Glycine max* (soybean) and that nonsynonymous mutations are present in conserved regions of POLD1 proteins in resistant parental varieties. Further, analysis of publicly available transcriptomics data and resequencing of cDNA clones from resistant cotton accessions revealed mutations within the *POLD1* transcript. Taking an even wider lens, as the majority of individuals within germplasm collections may not have been specifically screened for response to geminivirus infection, we sought to evaluate variation found within the POLD1 protein from whole-genome resequencing projects of diverse panels of plant species, positing that there may be untapped sources for new geminivirus resistance alleles found within these datasets. We analyzed the POLD1 sequence from over 7,000 plant accessions across 10 genera from both dicots and monocots, and here, we report several additional mutations within *POLD1* of species with no known geminivirus phenotype, suggesting possible new sources of geminivirus resistance alleles. Taken together, the analyses presented here show that variation within *POLD1* exists in diverse plant species, and in several cases, correlates with geminivirus resistance.

## Methods

### QTL, whole-genome resequencing, and sequencing sources

#### Cassava

Kompetitive allele-specific PCR (KASP) markers M3 and M7 used to identify the CMD2 QTL and the position of single nucleotide polymorphisms in *MePOLD1* in cassava mosaic resistant accessions were previously described (Lim et al. 2022). Genes between the markers were from the annotated haplotype-resolved genome assembly of cassava accession TME204 (Qi et al. 2022). Variant data within *MePOLD1* for 241 accessions were identified from the Cassava haplotype map (Ramu et al. 2017).

#### Tomato

Markers snp417 and sl_8774 used in identifying the *Ty-6* QTL are from (Gill et al. 2019). Annotation from version SL3.0 of the *Solanum lycopersicum* genome provided the list of genes between the markers. Position and identity of the SNP in *SlPOLD1* (Solyc10g081250) in geminivirus-resistant tomato is from Shen et al. (2025). Variation in *SlPOLD1* was identified from whole-genome resequencing of a diverse panel of 150 accessions of tomato (100 Tomato Genome Sequencing Consortium et al. 2014). Full-length cDNA of *POLD1* from *Solanum chilense accession* LA2779 was amplified from RNA extracted from young leaf tissue using the Spectrum Plant Total RNA Kit from Sigma-Aldrich, following the manufacturer's guidelines. RNA was treated with DNase I enzyme from New England Biolabs and reverse transcribed into cDNA using SuperScript II Reverse Transcriptase from Thermo Fisher Scientific. A targeted region of the resulting *POLD1* cDNA that encoded residues 502 to 732 of the ScPOLD1 protein, which encompasses the key N-terminal, palm, and finger motifs containing known resistance-related mutations, was amplified using primers TomPolD1roi 11F and TomPolD1roi 11R (Supplemental Table 1). All nucleic acids were quantified using a Nanodrop 2000 Spectrophotometer. Sanger sequencing of the targeted PCR product was performed at GENEWIZ (Azenta Life Sciences) using primer TomPolD1roi 11F. Seeds were acquired from The C.M. Rick Tomato Genetics Resource Center.

#### Cotton

The genome for *Gossypium hirsutum* accession TM-1, version 2 (Chen et al. 2020 ), was downloaded from Phytozome.com and indexed with hisat2 (v2.2.1) (Kim et al. 2019) and bowtie2 (v2.5.3) (Langmead and Salzberg 2012). Transcriptomic data for accession Mac7 were downloaded from SRA record SRR3067747, BioProject PRJNA307071, and aligned to the hisat2 index. Whole-genome sequencing data was downloaded for accession NN-3 (BioProject PRJNA901674; SRR22581629) (Han et al. 2024) and aligned to the bowtie2 index. The polymorphism identified in the whole genome sequencing in NN-3 (PI 665058) was confirmed by Sanger sequencing of full-length *GhPOLD1* cDNA, from seeds acquired from United States Department of Agriculture - Agricultural Research Service (USDA-ARS) Germplasm Resources Information Network (GRIN). Briefly, RNA was extracted from a young cotton leaf using the Spectrum Plant Total RNA kit (Sigma-Aldrich), and cDNA was synthesized using SuperScript III First-Strand Synthesis System (Thermo Fisher Science) following the manufacturer's instructions. As cotton encodes 2 copies of *POLD1*, Gohir.A13G139100 and Gohir.D13G143900, we used specific primers to amplify the full-length transcripts separately: primers 2F and 3560-R for Gohir.D13G143900 and primers 2F and 3606-R for Gohir.A13G139100 (Supplemental Table 1). Resultant PCR products were gel purified using the Zymoclean Gel DNA Recovery Kit (Zymo Research), and sequencing reactions for both genes were submitted for sequencing to GENEWIZ (Azenta Life Sciences) for Sanger sequencing with primer 1389F to generate sequence data for residues 482 to 689 of the GhPOLD1 protein sequence, which encompasses the key N-terminal, palm, and finger motifs that contain known resistance mutations.

#### Soybean

Markers Satt_322 and Sat_076 were previously identified as flanking the QTL for resistance against the geminivirus mung bean yellow mosaic India virus (MYMIV) in soybean (Rani et al. 2017; Khosla et al. 2021). An additional geminivirus resistance QTL study identified markers Gm06_11711313 to Gm06_14509272 (Mandahal et al. 2025). Annotation from version Wm82.a2.v1 of *Gl. max* Williams 82 was used to identify genes between the markers (Schmutz et al. 2010). Seeds for the geminivirus-resistant accession PI 171443 were acquired from USDA-ARS GRIN for sequencing of Glyma.06G155300. Briefly, a 2-step approach was used to identify and confirm polymorphisms in Glyma.06G155300. First, RNA was isolated from young leaf tissue using a Direct-zol RNA Miniprep kit (Zymo Research) and cDNA synthesis was performed using 1ug of RNA and SuperScript III First-Strand Synthesis System (Thermo Fisher Science), following the manufacturer's recommendations. Primers PG P44 and PG P45 (Supplemental Table 1) were used to amplify a targeted region of the cDNA corresponding to residues 502 to 696 of the GmPOLD1 protein sequence, which encompasses the key N-terminal, palm, and finger motifs that contain known resistance mutations. This targeted PCR amplicon was purified using PureLink PCR Purification Kit (Thermo Fisher Scientific) before sending out for Sanger sequencing by Azenta Life Sciences using primer PGP44. Nucleic acids were quantified using a Nanodrop 2000 Spectrophotometer and a Qubit Fluorometer. Results from this initial round of sequencing identified a mutation corresponding to residue Y515; however, the primers used for PCR amplification could not discriminate between the *POLD1* copy found on chromosome Gm06 from the one on chromosome Gm04. Thus, we designed a second set of primers based on the respective genomic sequences to separately amplify and sequence the exon containing the mutation from both Glyma.06G155300 and Glyma.04G210700 to confirm which copy contained the mutation. Genomic DNA was isolated from Pl 171443 using the DNeasy Plant Kit (QIAGEN), and primers PG P54 and PG P55 were used to amplify the region from Glyma.06G155300, while primers PG P56 and PG P57 were used to amplify the region in Glyma.04G210700 (Supplemental Table 1). Following PCR product clean-up by the PureLink PCR Purification Kit (Thermo Fisher Science), the forward primer of each pair was included with the purified product to GENEWIZ (Azenta Life Sciences) for Sanger sequencing. Additionally, variation within *Gm06POLD1* (Glyma.06G155300) was identified from the haplotype map variant dataset (GmHapMap) (Torkamaneh et al. 2021).

#### Squash

Markers S7_3,901,603-1, S7_4,017,657, and S7_4,089,911 were previously identified in the *Cu. moschata* (Duchesne ex Poir.) breeding line AVPU1426 as flanking the QTL for resistance against squash leaf curl China virus (SLCCNV) and ToLCNDV (Schafleitner et al. 2024). KASP markers Cmo3914729 and Cmo4018182 were previously identified in *Cu. moschata* as flanking a QTL for resistance to SLCCNV and ToLCNDV, and whole-genome sequencing of resistant (PVR-1343) and susceptible (P-135) parents as well as bulked resistant F1 and bulked susceptible F1 progeny was accessed from BioProject PRJNA842505 (Verma et al. 2023b). The *Cu. moschata* variety Rifu (v1) genome (Sun et al. 2017) was accessed from the Cucurbit Genomics Database (Yu et al. 2023; http://cucurbitgenomics.org) and indexed for alignment of data from PRJNA842505 using bowtie2 (v2.5.3) (Langmead and Salzberg 2012).

#### Cucumber

Variation within *CsPOLD1* (CsGy6G025560; Gy14 v2) was isolated from the core suite of SNPs of the 1,234 accessions of *Cucumis sativus* in the US National Plant Germplasm System (Wang et al. 2018).

#### Arabidopsis

Sanger sequencing of the ethyl methanesulfonate mutant *gis-5* (Iglesias et al. 2015) was used to confirm the presence of the homozygous mutation in *AtPOLD1* (AT5G63960) using the TAIR10 genome sequence as a reference (Reiser et al. 2024). Briefly, genomic DNA, isolated using the cetrimonium bromide protocol, was used as a template for PCR amplification of the exon containing the A707 residue by primers 660-gis5_geno_FW and 660-gis5_geno_RV (Supplemental Table 1). The resulting PCR product was cleaned using the PureLink PCR Purification Kit (Thermo Fisher Scientific), DNA quality and concentration was determined using the dsDNA Broad Range kit and a Qubit fluorometer (Thermo Fisher Scientific), and sequenced at Genewiz (Azenta Life Sciences) using primer 660-gis5_geno_RV. Variation within *AtPOLD1* was identified from 1,135 accessions of *Arabidopsis thaliana* from 1001 genomes project (1001 Genomes Consortium 2016).

#### Rice bean

Variation from whole-genome sequencing of a diversity panel of 353 accessions of *Vigna umbellata* was accessed at www.ricebeanportal.com (Francis et al. 2023) by downloading polymorphisms within *VuPOLD1* (Vu_09327).

#### Maize

Variation in *ZmPOLD1* (Zm00001eb201340; genome version: Zm-B73-REFERENCE-NAM-5.0) was isolated from the standardized variant calls in the MaizeGDB 2024 High Coverage dataset of 1,498 diverse maize accessions (Andorf et al. 2025) available at MaizeGDB (Woodhouse et al. 2021).

#### Wheat

Sequencing of 890 diverse wheat accessions as part of the 1000 wheat exome project (He et al. 2019) generated variant data using the reference wheat genome International Wheat Genome Sequencing Consortium RefSeq v1.0. Variation within the 3 genomic copies of *TaPOLD1* (TraesCS4A02G220100, TraesCS4B02G124700, TraesCS4D02G094700) was determined from the version 2.1 genome (Vasudevan and Cloutier 2023).

#### Medicago

Variation in *MtPOLD1* (Medtr3g096525) was identified from whole-genome resequencing data generated for 226 accessions of *Medicago truncatula* (Stanton-Geddes et al. 2013) using coordinates from the Mt4.0 genome assembly (Tang et al. 2014).

#### Pigeon pea

The first generation haplotype map for *Cajanus* species included whole-genome resequencing of 20 accessions (Kumar et al. 2016) and variation in *Caj. cajan POLD1* (C.cajan_04111) was extracted using genome version v1.1 coordinates.

#### Watermelon

Two studies generated resequencing data of an overlapping set of 414 (Guo et al. 2019) and 547 accessions (Wu et al. 2023) of *Citrullus* species. Variation within *ClPOLD1* (Cla97C01G014110) was determined using the coordinates of the v2.5 genome assembly.

The program snpEff was used to annotate and predict the effects of variation in *POLD1* (Cingolani et al. 2012). To standardize the analysis, the position of mutations from all noncassava species were converted to coordinates in the cassava POLD1 protein sequence (MePOLD1; Manes.12G077400). To do this, the protein sequence of POLD1 for each species was aligned to MePOLD1 using the command line version of MAFFT (v7.505) (Katoh et al. 2019). The fasta-formatted alignment was then used as the input to a custom Perl script along with the list of residues identified from the snpEff analysis for each species to generate the identity and position of the same residues in the MePOLD1 sequence (Supplementary File 1). Positions where the identity of the amino acid was not conserved in cassava were discarded. For the conserved residues, SIFT (https://sift.bii.a-star.edu.sg) (Ng and Henikoff 2003) was used to predict the effect of mutations on the function of POLD1, using a SIFT score cutoff of 0.06 and a sequence conservation score <3.10 to identify mutations predicted to not be tolerated. The SIFT-generated multiple sequence alignment file is available in Supplementary File 2.

### Figure preparation

**Fig. 1.**
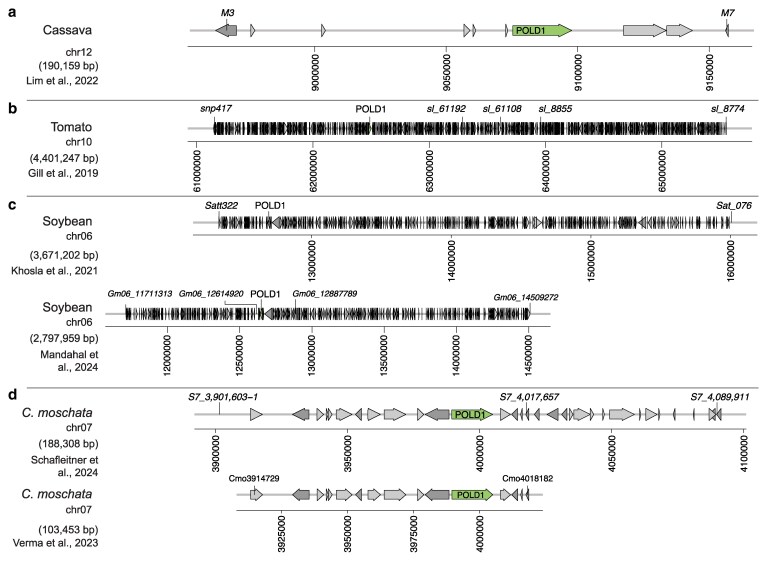
Geminivirus resistance QTLs previously described in a) cassava, b) tomato, c) soybean, and d) squash. The location of key markers is labeled, and genes within the QTL region are either in light gray (forward strand) or dark gray (reverse strand). The location of the POLD1 gene is labelled. The size of the QTL reported in the corresponding citation is in parentheses. The 2 QTL plots from soybean are drawn to the same scale; similarly, the 2 QTL plots for *Cu. moschata* are at the same scale to one another.

**Fig. 2.**
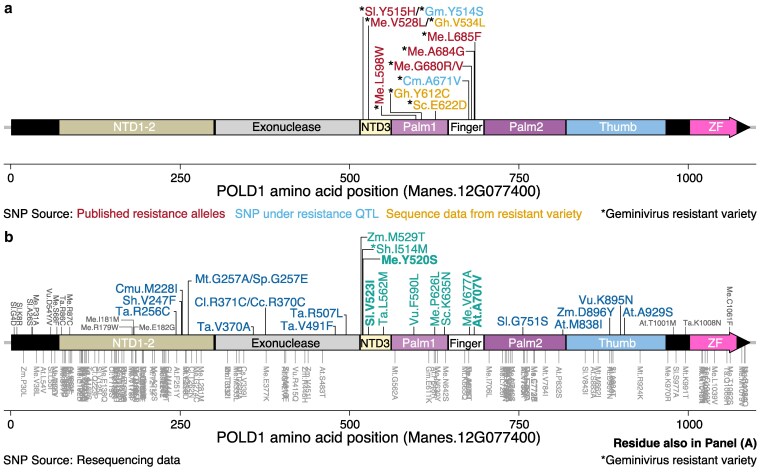
Location of mutations affecting the POLD1 protein in diverse plant species. a) Mutations in *POLD1* in geminivirus-resistant plant varieties. Red: positions affected by mutations identified as resistance alleles underlying resistance in cassava and tomato; light blue: positions affected by mutations found in *POLD1* present under geminivirus resistance QTLs in soybean and squash; orange: positions affected by mutations found in public sequencing data for resistant cotton and through targeted sequencing of the resistant accession *S. chilense* LA2779. All mutations are found within the N-terminal domain, catalytic center of the palm motif, and the finger motifs. b) Mutations identified in POLD1 from allele mining of over 7,000 varieties from 10 different genera, analyzed for predicted effect on function using SIFT. Mutations above the POLD1 diagram: SNPs predicted to negatively affect protein function; in blue and teal are high confidence SNPs and in gray are low confidence mutations. All mutations below the diagram are predicted to be tolerated by the POLD1 protein. Teal mutations are found in the same areas of the protein structure as all mutations in a); bolded mutations are at a position also found in a). For both a) and b), each SNP is labeled with a 2-letter code for the source species and coordinates for the position within the POLD1 protein sequence of the source species. Mutations found in geminivirus-resistant accessions are indicated with an asterisk (*). At, *A. thaliana*; Cc, *Caj. cajan*; Cl, *Citrullus lanatus*; Cm, *Cu. moschata*; Cmu, *Citrullus mucosospermus*; Gh, *G. hirsutum*; Gm, *Gl. max*; Me, *Manihot esculenta*; Mt, *Me. truncatula*; Sh, *S. huaylasense f. glabratum*; Sl, *S. lycopersicum*; Sp, *Solanum peruvianum*; Ta, *Triticum aestivum*; Vu, *V. umbellata*; Zm, *Z. mays*; Sc, *S. chilense*; Sc, *S. chmielewskii.*

Graphical representations of genes between the QTL markers described above were created using the R packages gggenes (Wilkins 2023) and ggplot2 (Wickham 2016). Similarly, gggenes and ggplot2 were used to generate a plot of the identified mutations in POLD1 along with the POLD1 protein domains and motifs. All plots were generated in R (v4.4.2) (R Core Team 2024) using RStudio (v2024.12.0) (RStudio Team 2020), and input files and R code for Figs. 1 and 2 are available on FigShare (https://doi.org/10.6084/m9.figshare.28585076).

The 3D structure of the human POLD1 catalytic subunit and template DNA (PDB ID: 6NTZ) was visualized in ChimeraX (v1.3) (Pettersen et al. 2021) to highlight the mutations from Fig. 2; an animated clip of this structure is available in Supplementary File 3.

## Results

### Investigation of previously reported geminivirus QTL for variation at POLD1

**Fig. 3.**
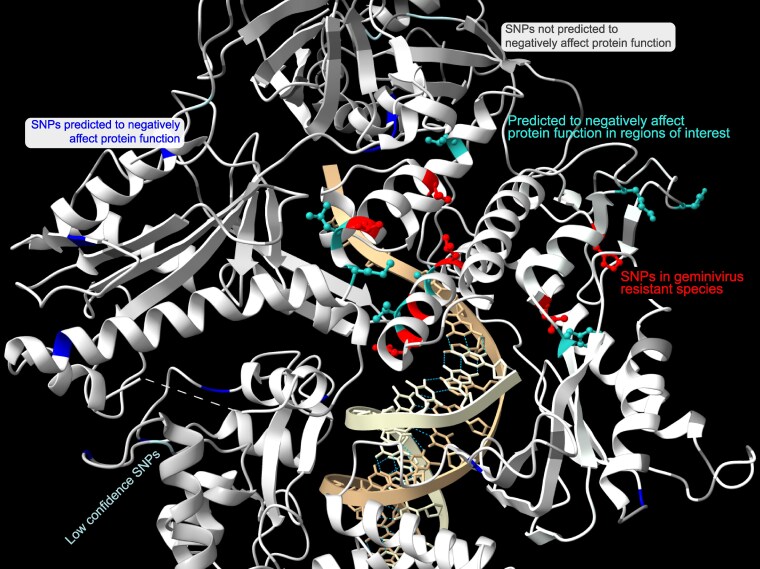
Model of the 3D protein structure of POLD1 from *Homo sapiens* (PDB ID: 6NTZ). Double-stranded DNA is in beige in the center of the protein structure. All residues from Fig. 2a are in red; teal residues from Fig. 2b are in teal; blue residues in Fig. 2b are in blue; low confidence residues from Fig. 2b are in light cyan; residues from Fig. 2b not predicted to affect protein structure are in dark gray.

The cassava *CMD2* (Rabbi et al. 2014) and tomato *Ty-6* loci (Gill et al. 2019) were previously characterized quantitative trait loci (QTLs) for geminivirus resistance (Fig. 1a, b). Recently, *DNA POLD1* was identified as the causal gene within the QTL for both cassava (Lim et al. 2022) and tomato (Shen et al. 2025). From cassava, 6 mutant alleles, reported in the heterozygous state, were identified: V528L, L598W, G680V, G680R, A684G, and L685F; all correlate with resistance, having an average Cassava Mosaic Disease (CMD) score ≤2 (Lim et al. 2022) (Fig. 2a). In tomato, the mutant allele, Y515H (Shen et al. 2025), was reported as a homozygous mutation affecting the same N-terminal domain alpha helix of the POLD1 protein structure as the V528L mutation in cassava (Figs. 2a and  3).

Multiple studies have identified a QTL for resistance to the geminivirus MYMIV on chromosome 6 in soybean (Rani et al. 2017; Khosla et al. 2021; Mandahal et al. 2025). *Gl. max* accessions resistant to MYMIV used in these studies were PI 171443, SL 525 (which has the same resistance as PI 171443), and SL 958 (a cross between resistant parents SL 525 × SL 706). An overlapping region on chromosome 6 was identified in all 3 studies, in which 1 of the 2 genomic copies of *GmPOLD1* is found (Fig. 1c). While whole-genome resequencing of PI 171443 (UPSM-534) and a susceptible variety “JS 335” identified SNPs across all chromosomes, neither raw sequencing data nor detailed variant data are available (Yadav et al. 2015). However, we obtained seed for accession PI 171443, prepared cDNA and determined the sequence of both the chromosome 6 and chromosome 4 *POLD1* transcripts. This revealed the presence of a homozygous A>C polymorphism at position Gm06:12,650,162 in Glyma.06G155300 (Wm82.a4.v1) resulting in a Y514S substitution, while the same residue in the chromosome 4 copy of *GmPOLD1* remained homozygous for tyrosine. This is the same residue that was recently identified as the source of geminivirus resistance in tomato (Shen et al. 2025) (Fig. 2a).

Multiple species of geminiviruses infect *Cu. moschata,* including SLCCNV and ToLCNDV. Two studies, using coinfection by these 2 viruses, identified the same region as a resistance QTL on chromosome 7 in 2 different resistant varieties: a 188-kb region in breeding line AVPU1426 and a 108-kb region in breeding line PVR-1343 (Verma et al. 2023b; Schafleitner et al. 2024) (Fig. 1d). Analysis of whole-genome resequencing data from PVR-1343 revealed the presence of a homozygous nonsynonymous SNP at position Cmo_Chr7:3,999,06 within *CmPOLD1* (CmoCh07G008460), which results in a A671V substitution that is not present in the susceptible variety P-135 (Supplementary Fig. 1). This alanine residue is found in the same finger motif of the POLD1 protein structure as multiple mutations identified in CMD-resistant cassava (Lim et al. 2022).

Multiple other QTLs from 13 different species, where the underlying resistance gene had not been identified, were also examined but did not contain *POLD1*. This indicates that a broad range of putative resistance genes remain to be identified and characterized (Table 1).

**Table 1.** Geminivirus resistance QTLs evaluated that do not contain POLD1.

Species	Virus	Chromosome	Ref
*G. hirsutum*	Cotton leaf curl virus	LG7 (A07), LG23 (D09), LG24 (D08)	Schoonmaker et al. (2023)
*G. hirsutum*	Cotton leaf curl virus	D01	Pathak et al. (2024)
*G. hirsutum*	Cotton leaf curl virus	C11 (A11), C19 (D05), C21 (D11)	Sattar et al. (2023)
*A. thaliana*	Beet curly top virus; cabbage leaf curl virus	Chr1	Reyes et al. (2017)
*Ca. annuum*	Beet curly top virus	Chr2, Chr3	Jimenez (2018)
*Ca. annuum*	Pepper yellow leaf curl virus	Chr1, Chr7, Chr12	Siddique et al. (2022)
*Cu. moschata*	ToLCNDV	Chr8	Sáez et al. (2020)
*C. melo*	ToLCNDV	Chr2, Chr11, Chr12	Sáez et al. (2017)
*C. melo*	Cucurbit yellow stunting disorder virus	Chr5	Tamang et al. (2021)
*C. sativus*	ToLCNDV	Chr2	Sáez et al. (2021)
*C. sativus*	ToLCNDV	Chr2	Koeda et al. (2024)
*Jatropha curcas*	Jatropha mosaic virus	Chr3, Chr4, Chr10	Kancharla et al. (2019)
*Momordica charantia*	Yellow mosaic disease	Chr3, Chr4, Chr5	Kaur et al. (2021)
*P. vulgaris*	Bean golden yellow mosaic virus	Chr03, Chr04, Chr07, Chr08	Soler-Garzón et al. (2021)
*P. vulgaris*	Beet curly top virus	Chr07	Soler-Garzón et al. (2023)
*Triticum aestivum*	Wheat dwarf virus	Chr6A, Chr1B	Buerstmayr and Buerstmayr (2023)
*T. aestivum*	Wheat dwarf virus	Chr1B, Chr1D, Chr2B, Chr3A, Chr3B, Chr4A, Chr4B, Chr5A, Chr6A, Chr7A, Chr7B	Pfrieme et al. (2022)
*V. mungo*	MYMV	LG10	Subramaniyan et al. (2022)
*V. mungo*	Mung bean yellow mosaic India virus	Chr2, Chr4, Chr6, Chr7, Chr8, Chr9	Pandey et al. (2024)
*V. mungo*	Mung bean yellow mosaic India virus	Chr2, Chr10	Vadivel et al. (2021)
*Vigna radiata*	MYMV	Chr04, Chr05, Chr06	Mathivathana et al. (2019)
*V. radiata*	MYMV	LG2, LG7	Alam et al. (2014)
*Z. mays*	Maize streak virus	Chr3, Chr7, Chr9	Ladejobi et al. (2018)
*Z. mays*	Maize streak virus	Chr1, Chr2, Chr3, Chr4, Chr5 and Chr7	Garcia-Oliveira et al. (2020)
*Z. mays*	Maize streak virus	Chr1	Nair et al. (2015)

### Investigation of previously reported geminivirus-resistant germplasm for variation at POLD1

Geminivirus-resistant varieties have been described from diverse plant species without defined QTL. In several such cases, publicly available sequencing data are available for the geminivirus-resistant varieties. As cotton is an allotetraploid, both the A-genome and D-genome encode an intact copy of the *POLD1* gene. Data from 2 different geminivirus-resistant cotton accessions were analyzed for variation in the A and D copies of *GhPOLD1* (Gohir.A13G139100 and Gohir.D13G143900). *G. hirsutum* accession Mac7 is a product of a joint USDA-Pakistan screening program to identify cotton germplasm with resistance to cotton leaf curl disease (CLCuD) (Zaidi et al. 2020). Analysis of available Mac7 transcriptomic data revealed a heterozygous SNP present in both copies of *GhPOLD1* at position A13:94,279,727 and D13:50,254,530 (Supplementary Fig. 2). Due to the high level of sequence identity between the 2 gene copies, 20% of the reads could definitively be assigned to the A13 copy, while the remaining 80% of reads matched both copies. This could indicate that instead of a heterozygous SNP in each *GhPOLD1* gene, one copy is homozygous for the alternative allele and the other is homozygous for the reference allele. Whole-genome resequencing of Mac7 was recently reported (Hussain et al. 2022); however, the genome assembly and raw data are unavailable and therefore we were not able to resolve the distribution of the V534L mutation on the A13 and/or D13 chromosomes. The identified nonsynonymous mutation resulted in a V534L mutation, the same mutation in CMD-resistant cassava varieties TME204 and TME419 as V528L (Fig. 2a) (Lim et al. 2022).

*G. hirsutum* accession NN-3 (PI 665058) is the result of a cross between S-12 (a high-yielding, CLCuD susceptible variety) and LRA-5166 (a variety resistant to the Multan strain of CLCuD) and demonstrates high tolerance to virus infection (Rahman and Zafar 2012). Analysis of whole-genome resequencing data for NN-3 (Han et al. 2024) revealed the presence of a homozygous missense mutation in *GhPOLD1* on chromosome D13 at position 50,254,103, while the A13 copy of *GhPOLD1* is homozygous for the reference allele at the comparable position 94,279,299 (Supplementary Fig. 3). The effect of this mutation was that both copies at D13:*GhPOLD1* code for a Y612C substitution, while the 2 A13 copies code for Y612. Analysis of cDNA sequences of *GhPOLD1* from each A and D genomes confirmed this distribution of alleles. The Y612 residue is found in the palm motif of POLD1 near to the L598W position identified in CMD-resistant cassava (Figs. 2a and 3).

The wild tomato accession *S. chilense* LA2779, which carries the dominant *Ty-3* allele of an RNA-dependent RNA polymerase gene that confers resistance to TYLCV, has also been reported to carry a second gene conferring incomplete resistance to TYLCV on chromosome 10 (Gill et al. 2019). Sequencing of full-length cDNA of *ScPOLD1* identified a heterozygous polymorphism, resulting in an E622D substitution affecting a residue within the palm motif of POLD1 (Fig. 2a).

### Investigating publicly available resequencing data for variation at POLD1 from diverse dicot and monocot germplasm

To identify additional putative resistance alleles among germplasm collections, we examined *POLD1* from whole-genome resequencing data for multiple plant species (irrespective of known geminivirus resistance phenotypes), identified all sequence variation affecting the POLD1 protein sequence, and assessed potential negative effects on protein function using SIFT (Ng and Henikoff 2003) (Table 2).

**Table 2.** Summary mutations analyzed from resequencing projects of diverse germplasm in both dicots and monocots.

Pos (a)	Ref AA	Alt AA	Mutation	Pos (b)	Source organism	Pred.	SIFT score	Seq conservation	Seq reported	Warning	Ref
5	G	D	Sl.G4D^a^	4	*S. lycopersicum*	Affect	0	4.32	2	LC	100 Tomato Genome Sequencing Consortium et al. (2014)
9	K	R	Sl.K8R^a^	8	*S. lycopersicum*	Affect	0	4.32	2	LC	100 Tomato Genome Sequencing Consortium et al. (2014)
19	P	L	Zm.P30L^b^	30	*Z. mays*	Tolerated	0.23	4.32	2	—	Andorf et al. (2025)
31	P	A	Me.P31A^a^	31	*Manihot esculenta*	Affect	0	4.32	2	LC	Ramu et al. (2017)
32	A	S	Sl.A26S^a^	26	*S. lycopersicum*	Affect	0	4.32	3	LC	100 Tomato Genome Sequencing Consortium et al. (2014)
38	V	L	Me.V38L^b^	38	*M. esculenta*	Tolerated	0.48	4.32	4	—	Ramu et al. (2017)
48	L	V	At.L54V^b^	54	Arabidopsis	Tolerated	0.1	4.05	5	—	1001 Genomes Consortium (2016)
59	D	Y	Vu.D54Y^a^	54	Rice bean	Affect	0	3.56	5	LC	Francis et al. (2023)
59	D	V	Vu.D54V^a^	54	Rice bean	Affect	0	3.56	5	LC	Francis et al. (2023)
60	L	I	Sl.L55I^b^	55	*S. lycopersicum*	Tolerated	0.99	3.54	7	—	100 Tomato Genome Sequencing Consortium et al. (2014)
65	A	V	Me.A65V^b^	65	*M. esculenta*	Tolerated	0.59	3.56	6	—	Ramu et al. (2017)
65	A	D	Mt.A59D^b^	59	*Me. truncatula*	Tolerated	0.65	3.56	6	—	*Stanton-Geddes* et al*. (2013)*
68	S	F	Me.S68F^a^	68	*M. esculenta*	Affect	0.02	3.54	7	LC	Ramu et al. (2017)
75	R	C	Ta.R86C^a^	86	Wheat_4D	Affect	0	3.32	16	LC	He et al. (2019)
77	S	P	Me.S77P^b^	77	*M. esculenta*	Tolerated	0.89	3.32	16	—	Ramu et al. (2017)
79	S	C	Me.S79C^b^	79	*M. esculenta*	Tolerated	0.2	3.33	14	—	Ramu et al. (2017)
79	S	P	At.S85P^b^	85	Arabidopsis	Tolerated	0.86	3.33	14	—	1001 Genomes Consortium (2016)
81	E	D	Me.E81D^b^	81	*M. esculenta*	Tolerated	0.65	3.32	16	—	Ramu et al. (2017)
86	S	F	Mt.S80F^b^	80	*Me. truncatula*	Tolerated	0.08	3.31	15	—	Stanton-Geddes et al. (2013)
87	R	C	Me.R87C^a^	87	*M. esculenta*	Affect	0.03	3.32	16	LC	Ramu et al. (2017)
88	S	T	Sl.S83T^b^	83	*S. lycopersicum*	Tolerated	0.28	3.31	15	—	100 Tomato Genome Sequencing Consortium et al. (2014)
90	I	V	At.I96V^b^	96	Arabidopsis	Tolerated	0.58	3.32	16	—	1001 Genomes Consortium (2016)
102	E	D	Me.E102D^b^	102	*M. esculenta*	Tolerated	0.29	3.32	16	—	Ramu et al. (2017)
104	H	N	Me.H104N^b^	104	*M. esculenta*	Tolerated	0.31	3.32	16	—	Ramu et al. (2017)
105	K	R	Me.K105R^b^	105	*M. esculenta*	Tolerated	0.52	3.31	15	—	Ramu et al. (2017)
106	E	V	Me.E106V^b^	106	*M. esculenta*	Tolerated	0.28	3.31	15	—	Ramu et al. (2017)
109	P	L	Ta.P121L^b^	121	Wheat_4A	Tolerated	0.21	3.12	14	—	He et al. (2019)
110	D	A	Me.D110A^b^	110	*M. esculenta*	Tolerated	0.76	3.09	15	—	Ramu et al. (2017)
110	D	N	Me.D110N^b^	110	*M. esculenta*	Tolerated	0.83	3.09	15	—	Ramu et al. (2017)
112	S	Y	Mt.S106Y^b^	106	*Me. truncatula*	Tolerated	0.07	3.07	16	—	Stanton-Geddes et al. (2013)
115	A	S	Ta.A127S^b^	127	Wheat_4D	Tolerated	0.45	3.09	16	—	He et al. (2019)
124	T	S	Cl.T119S^b^	119	Watermelon	Tolerated	0.08	3.06	17	—	Guo et al. (2019)
130	V	I	Sl.V125I^b^	125	*S. lycopersicum*	Tolerated	0.21	3.06	17	—	100 Tomato Genome Sequencing Consortium et al. (2014)
138	E	Q	Me.E138Q^b^	138	*M. esculenta*	Tolerated	0.56	3.06	17	—	Ramu et al. (2017)
149	M	L	Cl.M144L^b^	144	Watermelon	Tolerated	0.14	3.06	17	—	Wu et al. (2023)
153	D	N	At.D159N^b^	159	Arabidopsis	Tolerated	0.84	3.06	17	—	1001 Genomes Consortium (2016)
155	S	P	Sl.S150P^b^	150	*S. lycopersicum*	Tolerated	0.25	3.06	17	—	100 Tomato Genome Sequencing Consortium et al. (2014)
156	R	G	Me.R156G^b^	156	*M. esculenta*	Tolerated	0.34	3.06	17	—	Ramu et al. (2017)
159	Q	H	Me.Q159H^b^	159	*M. esculenta*	Tolerated	0.4	3.32	16	—	Ramu et al. (2017)
164	R	K	Cc.R158K^b^	158	Pigeon pea	Tolerated	1	3.16	15	—	Andorf et al. (2025)
167	E	D	Zm.E179D^b^	179	*Z. mays*	Tolerated	0.63	3.07	16	—	Andorf et al. (2025)
167	E	A	Mt.E161K^b^	161	*Me. truncatula*	Tolerated	0.75	3.07	16	—	Stanton-Geddes et al. (2013)
167	E	K	Zm.E179A^b^	179	*Z. mays*	Tolerated	0.91	3.07	16	—	Andorf et al. (2025)
178	V	I	Me.V178I^b^	178	*M. esculenta*	Tolerated	0.42	3.32	15	—	Ramu et al. (2017)
179	R	W	Me.R179W^a^	179	*M. esculenta*	Affect	0.03	3.32	15	LC	Ramu et al. (2017)
180	R	S	Vu.R175S^b^	175	Rice bean	Tolerated	0.65	3.32	15	—	Francis et al. (2023)
181	I	M	Me.I181M^a^	181	*M. esculenta*	Affect	0.01	3.32	16	LC	Ramu et al. (2017)
182	E	G	Me.E182G^a^	182	*M. esculenta*	Affect	0.01	3.32	16	LC	Ramu et al. (2017)
184	V	A	Me.V184A^b^	184	*M. esculenta*	Tolerated	0.29	3.07	16	—	Ramu et al. (2017)
185	Q	L	Sl.Q180L^b^	180	*S. lycopersicum*	Tolerated	0.21	3.07	16	—	100 Tomato Genome Sequencing Consortium et al. (2014)
185	Q	R	Me.Q185R^b^	185	*M. esculenta*	Tolerated	0.54	3.07	16	—	Ramu et al. (2017)
194	Q	P	Mt.Q189P^b^	189	*Me. truncatula*	Tolerated	0.27	3.06	17	—	Stanton-Geddes et al. (2013)
194	Q	E	Me.Q194E^b^	194	*M. esculenta*	Tolerated	0.33	3.06	17	—	Ramu et al. (2017)
195	Q	P	Cl.Q223K^b^	223	Watermelon	Tolerated	0.17	3.06	17	—	Wu et al. (2023)
195	Q	K	Cl.Q223P^b^	223	Watermelon	Tolerated	0.47	3.06	17	—	Wu et al. (2023)
196	P	L	Me.P196L^b^	196	*M. esculenta*	Tolerated	0.3	3.06	17	—	Ramu et al. (2017)
209	T	I	At.T215I^b^	215	Arabidopsis	Tolerated	0.16	3.06	17	—	1001 Genomes Consortium (2016)
212	A	S	Me.A212S^b^	212	*M. esculenta*	Tolerated	0.84	3.06	17	—	Ramu et al. (2017)
224	I	L	Me.I224L^b^	224	*M. esculenta*	Tolerated	0.73	3.07	16	—	Ramu et al. (2017)
225	D	E	Sl.D220E^b^	220	*S. lycopersicum*	Tolerated	1	3.31	15	—	100 Tomato Genome Sequencing Consortium et al. (2014)
227	L	V	Mt.L222V^b^	222	*Me. truncatula*	Tolerated	0.44	3.31	15	—	Stanton-Geddes et al. (2013)
229	M	T	Me.M229T^b^	229	*M. esculenta*	Tolerated	0.33	3.32	16	—	Ramu et al. (2017)
229	M	V	Sl.M224V^b^	224	*S. lycopersicum*	Tolerated	1	3.32	16	—	100 Tomato Genome Sequencing Consortium et al. (2014)
233	M	I	Cmu.M228I^b^	228	*Citrullus mucosospermus*	Tolerated	0.27	3.32	16	—	*Wu* et al*. (2023)*
244	R	C	Ta.R256C^c^	256	Wheat_4D	Affect	0	3.06	17	—	He et al. (2019)
245	F	Y	At.F251Y^b^	251	Arabidopsis	Tolerated	0.24	3.06	17	—	1001 Genomes Consortium (2016)
252	V	F	Sl.V247F^c^	247	*S. lycopersicum*	Affect	0.03	3.06	17	—	100 Tomato Genome Sequencing Consortium et al. (2014)
253	G	V	Cl.G248V^c^	248	Watermelon	Affect	0.02	3.06	17	—	Guo et al. (2019)
258	E	D	Mt.E253D^b^	253	*Me. truncatula*	Tolerated	0.53	3.06	17	—	Stanton-Geddes et al. (2013)
259	V	I	Sl.V254I^b^	254	*S. lycopersicum*	Tolerated	0.57	3.06	17	—	100 Tomato Genome Sequencing Consortium et al. (2014)
262	G	A	Mt.G257A^c^	257	*Me. truncatula*	Affect	0.02	3.06	17	—	Stanton-Geddes et al. (2013)
262	G	E	Sp.G257E^c^	257	*S. peruvianum*	Affect	0.03	3.06	17	—	100 Tomato Genome Sequencing Consortium et al. (2014)
267	T	N	Cl.T262N^b^	262	Watermelon	Tolerated	0.51	3.06	17	—	Guo et al. (2019)
274	G	C	Me.G274C^b^	274	*M. esculenta*	Tolerated	1	3.06	17	—	Ramu et al. (2017)
281	L	M	Me.L281M^b^	281	*M. esculenta*	Tolerated	0.12	3.06	17	—	Ramu et al. (2017)
320	T	I	Mt.T315I^b^	315	*Me. truncatula*	Tolerated	0.19	3.06	17	—	Stanton-Geddes et al. (2013)
320	T	I	Zm.T332I^b^	332	*Z. mays*	Tolerated	0.19	3.06	17	—	Andorf et al. (2025)
333	M	L	Me.M333L^b^	333	*M. esculenta*	Tolerated	0.8	3.06	17	—	Ramu et al. (2017)
337	D	A	Mt.D332A^b^	332	*Me. truncatula*	Tolerated	0.85	3.06	17	—	Stanton-Geddes et al. (2013)
344	V	I	Cs.V339I^b^	339	Cucumber	Tolerated	0.35	3.06	17	—	Wang et al. (2018)
358	V	A	Ta.V370A^c^	370	Wheat_4D	Affect	0.01	3.06	17	—	He et al. (2019)
376	R	C	Cc.R370C^c^	370	Pigeon pea	Affect	0.06	3.06	17	—	Kumar et al. (2016)
376	R	C	Cl.R371C^c^	371	Watermelon	Affect	0.06	3.06	17	—	Wu et al. (2023)
377	E	K	Me.E377K^b^	377	*M. esculenta*	Tolerated	0.81	3.06	17	—	Ramu et al. (2017)
404	G	A	At.G427A^b^	427	Arabidopsis	Tolerated	0.36	3.06	17	—	1001 Genomes Consortium (2016)
406	A	G	Zm.A418E^b^	418	*Z. mays*	Tolerated	0.38	3.06	17	—	Andorf et al. (2025)
406	A	E	Sl.A401G^b^	401	*S. lycopersicum*	Tolerated	1	3.06	17	—	100 Tomato Genome Sequencing Consortium et al. (2014)
420	R	Q	Vu.R415Q^b^	415	Rice bean	Tolerated	0.59	3.06	17	—	Francis et al. (2023)
434	R	H	Zm.R446H^b^	446	*Z. mays*	Tolerated	0.12	3.06	17	—	Andorf et al. (2025)
439	V	I	Zm.V451I^b^	451	*Z. mays*	Tolerated	0.65	3.06	17	—	Andorf et al. (2025)
460	S	T	At.S483T^b^	483	Arabidopsis	Tolerated	0.26	3.06	17	—	1001 Genomes Consortium (2016)
479	V	F	Ta.V491F^c^	491	Wheat_4D	Affect	0.01	3.06	17	—	He et al. (2019)
495	R	L	Ta.R507L^c^	507	Wheat_4D	Affect	0	3.06	17	—	He et al. (2019)
517	M	T	Zm.M529T^d^	529	*Z. mays*	Affect	0.01	3.06	17	—	Andorf et al. (2025)
519	I	M	Sh.I514M^d^	514	*S. huaylasense*	Affect	0.06	3.06	17	—	100 Tomato Genome Sequencing Consortium et al. (2014)
520	Y	S	Me.Y520S^d^	520	*M. esculenta*	Affect	0.06	3.06	17	—	Ramu et al. (2017)
528	V	L	Me.V528L^d^	528	*M. esculenta*	Affect	0.03	3.06	17	—	Ramu et al. (2017)
528	V	I	Sl.V523I^d^	523	*S. lycopersicum*	Affect	0.04	3.06	17	—	100 Tomato Genome Sequencing Consortium et al. (2014)
550	L	M	Ta.L562M^d^	562	Wheat_4A	Affect	0.06	3.06	17	—	He et al. (2019)
567	G	A	Mt.G562A^b^	562	*Me. truncatula*	Tolerated	1	3.06	17	—	Stanton-Geddes et al. (2013)
595	F	L	Vu.F590L^d^	590	Rice bean	Affect	0	3.06	17	—	Francis et al. (2023)
598	L	W	Me.L598W^d^	598	*M. esculenta*	Affect	0	3.06	17	—	Ramu et al. (2017)
617	E	K	Gm.E611K^b^	611	Soybean	Tolerated	0.71	3.32	16	—	Torkamaneh et al. (2021)
625	P	R	At.P648R^b^	648	Arabidopsis	Tolerated	0.24	3.09	16	—	1001 Genomes Consortium (2016)
626	P	L	Me.P626L^d^	626	*M. esculenta*	Affect	0.06	3.09	16	—	Ramu et al. (2017)
630	N	Y	Me.N630Y^b^	630	*M. esculenta*	Tolerated	1	3.06	17	—	Ramu et al. (2017)
640	K	N	Sc.K635N^d^	635	*S. chmielewskii*	Affect	0.03	3.06	17	—	*100 Tomato Genome Sequencing Consortium* et al*. (2014)*
642	N	S	Me.N642S^b^	642	*M. esculenta*	Tolerated	1	3.06	17	—	Ramu et al. (2017)
670	K	Q	Mt.K665Q^b^	665	*Me. truncatula*	Tolerated	0.1	3.06	17	—	Stanton-Geddes et al. (2013)
676	A	T	Me.A676T^b^	676	*M. esculenta*	Tolerated	0.41	3.06	17	—	Ramu et al. (2017)
676	A	T	Ta.A688T^b^	688	Wheat_4A	Tolerated	0.41	3.06	17	—	He et al. (2019)
677	V	A	Me.V677A^d^	677	*M. esculenta*	Affect	0.01	3.06	17	—	Ramu et al. (2017)
680	G	R	Me.G680R^d^	680	*M. esculenta*	Affect	0	3.06	17	—	Ramu et al. (2017)
680	G	V	Me.G680V^d^	680	*M. esculenta*	Affect	0.01	3.06	17	—	Ramu et al. (2017)
684	A	V	At.A707V^d^	707	Arabidopsis	Affect	0.01	3.06	17	—	Iglesias et al. (2015)
684	A	G	Me.A684G^d^	684	*M. esculenta*	Affect	0.03	3.06	17	—	Ramu et al. (2017)
685	L	F	Me.L685F^d^	685	*M. esculenta*	Affect	0.06	3.06	17	—	Ramu et al. (2017)
706	I	L	Me.I706L^b^	706	*M. esculenta*	Tolerated	1	3.06	17	—	Ramu et al. (2017)
726	L	I	Me.L726I^b^	726	*M. esculenta*	Tolerated	0.61	3.06	17	—	Ramu et al. (2017)
730	K	N	Me.K730N^b^	730	*M. esculenta*	Tolerated	0.33	3.06	17	—	Ramu et al. (2017)
732	T	I	Me.T732I^b^	732	*M. esculenta*	Tolerated	0.08	3.06	17	—	Ramu et al. (2017)
732	T	P	Zm.T744P^b^	744	*Z. mays*	Tolerated	0.19	3.06	17	—	Andorf et al. (2025)
735	G	E	At.G758E^b^	758	Arabidopsis	Tolerated	0.08	3.32	16	—	1001 Genomes Consortium (2016)
735	G	A	Me.G735A^b^	735	*M. esculenta*	Tolerated	0.08	3.32	16	—	Ramu et al. (2017)
738	E	K	Cs.E733K^b^	733	Cucumber	Tolerated	0.72	3.32	16	—	Wang et al. (2018)
740	N	S	Me.N740S^b^	740	*M. esculenta*	Tolerated	0.22	3.06	17	—	Ramu et al. (2017)
756	G	S	Sl.G751S^c^	751	*S. lycopersicum*	Affect	0.03	3.06	17	—	100 Tomato Genome Sequencing Consortium et al. (2014)
757	V	I	At.V780I^b^	780	Arabidopsis	Tolerated	0.16	3.06	17	—	1001 Genomes Consortium (2016)
758	P	A	Sl.P753A^b^	753	*S. lycopersicum*	Tolerated	0.67	3.06	17	—	100 Tomato Genome Sequencing Consortium et al. (2014)
762	E	A	Me.E762A^b^	762	*M. esculenta*	Tolerated	0.14	3.06	17	—	Ramu et al. (2017)
762	E	D	Cc.E756D^b^	756	Pigeon pea	Tolerated	0.14	3.06	17	—	Kumar et al. (2016)
772	E	D	Me.E772D^b^	772	*M. esculenta*	Tolerated	0.87	3.06	17	—	Ramu et al. (2017)
773	C	R	Me.C773R^b^	773	*M. esculenta*	Tolerated	0.35	3.06	17	—	Ramu et al. (2017)
773	C	Y	Me.C773Y^b^	773	*M. esculenta*	Tolerated	1	3.06	17	—	Ramu et al. (2017)
789	V	I	Mt.V784I^b^	784	*Me. truncatula*	Tolerated	0.27	3.06	17	—	Stanton-Geddes et al. (2013)
809	P	S	At.P832S^b^	832	Arabidopsis	Tolerated	0.13	3.06	17	—	1001 Genomes Consortium (2016)
815	M	I	At.M838I^c^	838	Arabidopsis	Affect	0.05	3.06	17	—	1001 Genomes Consortium (2016)
848	V	I	Sl.V843I^b^	843	*S. lycopersicum*	Tolerated	0.46	3.06	17	—	100 Tomato Genome Sequencing Consortium et al. (2014)
860	S	A	At.S883A^b^	883	Arabidopsis	Tolerated	0.32	3.06	17	—	1001 Genomes Consortium (2016)
867	M	I	Mt.M862I^b^	862	*Me. truncatula*	Tolerated	1	3.06	17	—	Stanton-Geddes et al. (2013)
884	D	Y	Zm.D896Y^c^	896	*Z. mays*	Affect	0.01	3.06	17	—	Andorf et al. (2025)
884	D	N	At.D907N^b^	907	Arabidopsis	Tolerated	0.21	3.06	17	—	1001 Genomes Consortium (2016)
887	V	I	At.V910I^b^	910	Arabidopsis	Tolerated	0.12	3.06	17	—	1001 Genomes Consortium (2016)
889	A	T	Sl.A884T^b^	884	*S. lycopersicum*	Tolerated	0.7	3.32	16	—	100 Tomato Genome Sequencing Consortium et al. (2014)
900	K	N	Vu.K895N^c^	895	Rice bean	Affect	0.04	3.06	17	—	Francis et al. (2023)
906	A	S	At.A929S^c^	929	Arabidopsis	Affect	0.05	3.06	17	—	1001 Genomes Consortium (2016)
929	R	K	Mt.R924K^b^	924	*Me. truncatula*	Tolerated	0.63	3.06	17	—	Stanton-Geddes et al. (2013)
970	K	R	Me.K970R^b^	970	*M. esculenta*	Tolerated	0.54	3.06	17	—	Ramu et al. (2017)
978	T	M	At.T1001M^a^	1001	Arabidopsis	Affect	0.01	3.32	16	LC	1001 Genomes Consortium (2016)
982	S	A	Sl.S977A^b^	977	*S. lycopersicum*	Tolerated	0.22	3.32	16	—	100 Tomato Genome Sequencing Consortium et al. (2014)
996	K	N	Mt.K991T^a^	991	*Me. truncatula*	Affect	0.05	3.32	16	LC	Stanton-Geddes et al. (2013)
996	K	T	Ta.K1008N^b^	1008	Wheat_4A	Tolerated	0.32	3.32	16	—	He et al. (2019)
1020	K	N	Mt.K1015N^b^	1015	*Me. truncatula*	Tolerated	0.27	3.3	14	—	Stanton-Geddes et al. (2013)
1020	K	R	Mt.K1015R^b^	1015	*Me. truncatula*	Tolerated	0.28	3.3	14	—	Stanton-Geddes et al. (2013)
1021	G	E	At.G1042E^b^	1042	Arabidopsis	Tolerated	0.42	3.31	15	—	1001 Genomes Consortium (2016)
1025	E	K	At.E1046K^b^	1046	Arabidopsis	Tolerated	0.24	3.31	15	—	1001 Genomes Consortium (2016)
1025	E	K	Sl.E1020K^b^	1020	*S. lycopersicum*	Tolerated	0.24	3.31	15	—	100 Tomato Genome Sequencing Consortium et al. (2014)
1028	C	Y	Ta.C1040Y^b^	1040	Wheat_4D	Tolerated	0.93	3.31	15	—	He et al. (2019)
1039	L	V	Me.L1039V^b^	1039	*M. esculenta*	Tolerated	0.46	3.3	14	—	Ramu et al. (2017)
1057	Q	H	Ta.Q1069H^b^	1069	Wheat_4D	Tolerated	0.06	3.3	14	—	He et al. (2019)
1061	C	F	Me.C1061F^a^	1061	*M. esculenta*	Affect	0	3.3	14	LC	Ramu et al. (2017)
1062	T	S	Me.T1062S^b^	1062	*M. esculenta*	Tolerated	0.9	3.3	14	—	Ramu et al. (2017)
1079	M	V	Me.M1079V^b^	1079	*M. esculenta*	Tolerated	0.15	3.3	14	—	Ramu et al. (2017)
1083	K	R	Sl.K1078R^b^	1078	*S. lycopersicum*	Tolerated	0.49	3.3	14	—	100 Tomato Genome Sequencing Consortium et al. (2014)
1084	R	Q	Me.R1084Q^b^	1084	*M. esculenta*	Tolerated	0.55	3.3	14	—	Ramu et al. (2017)

Pos(a): Position in the cassava POLD1 (Manes.12G077400) protein. Pos(b): Position in the POLD1 protein of the source species.

LC, low confidence.

^a^Low confidence mutations (medium gray residues in Fig. 2b).

^b^Predicted to be tolerated by the POLD1 protein (light gray residues in Fig. 2b).

^c^High confidence mutations predicted to negatively affect protein function that are found in other domains than the mutations in Fig. 2a (navy blue residues in Fig. 2b).

^d^High confidence mutations predicted to negatively affect protein function that are found in the same domains as all mutations in Fig. 2a (teal residues in Fig. 2b).

#### Tomato

Variation in *POLD1* from 150 accessions of tomato (100 Tomato Genome Sequencing Consortium et al. 2014) revealed 6 nonsynonymous mutations predicted to negatively impact protein function (Table 2). Of these, 3 mutations were of particular interest as they are located within the conserved regions described above: Sh.I519M, Sl.V528I, and Sc.K640M (Table 2; Fig. 2b). The V523I mutation, at the same residue as the V528L mutation in cassava and V534L mutation in cotton (Fig. 2a), was found in *S. lycopersicum* LA1479 in a heterozygous state, while the K63N mutation was found in 2 accessions of *Solanum chmielewskii* in a homozygous state. To date, a geminivirus phenotype has not been described for any of these accessions. The I514M mutation, identified in *Solanum huaylasense* accessions LA1364 and LA1365, was in the heterozygous state and both accessions were previously described as symptomless for tomato yellow leaf curl disease following both field assays of natural geminivirus infection by whiteflies and *Agrobacterium*-mediated inoculation (Yan et al. 2018).

#### Soybean

The Haplotype Map (GmHapMap) project generated whole-genome resequencing data for 1,007 accessions of *Gl. max* (Torkamaneh et al. 2021). Two nonsynonymous SNPs were identified in the chromosome 6 copy of *GmPOLD1* (Glyma.06G155300) (Table 2): a Y514S mutation in accession PI 567383 and an E611K mutation in 5 accessions (PI089772, PI468915, PI548415, PI567357, and PI567611). The Y514S mutation is the same mutation identified in geminivirus-resistant *Gl. max* accession PI 171443 (Figs. 1c and 2a), the source of resistance in multiple geminivirus QTL analyses (Rani et al. 2017; Khosla et al. 2021; Mandahal et al. 2025), suggesting that PI 567383 likely has resistance to disease caused by MYMIV. There are currently no published data indicating the response to geminivirus infection for any of the 7 accessions with variant POLD1 sequences identified here.

#### Rice bean

Querying variation data from a diversity panel of 353 accessions of rice bean, *V. umbellata*, identified 5 nonsynonymous mutations in *VuPOLD1* (Vu_09327) (Francis et al. 2023). Of the 2 predicted to negatively impact protein function (Table 2), F590L was present within the catalytic palm motif, near the cassava L598F mutation identified in CMD-resistant varieties of cassava (Lim et al. 2022) (Figs. 2b and 3). The mutation was identified as heterozygous in accession GP-200 (EC934360 or JP 239946); no published data are available on the response to infection by MYMIV for this accession.

#### Arabidopsis

Variation data from resequencing of 1,135 genomes of Arabidopsis revealed 32 missense mutations in *AtPOLD1* (1001 Genomes Consortium 2016), 2 of which were determined to negatively impact protein function (Table 2). A separate study identified a temperature-sensitive allele (*gis-5*) of *AtPOLD1*, as a homozygous polymorphism resulting in a A707V substitution in the protein sequence (Iglesias et al. 2015). A mutation affecting this alanine residue was also identified in geminivirus-resistant cassava as A684G as deleterious (Table 2; Fig. 2a, b) and was found within the same finger motif of POLD1 as other geminivirus resistance alleles (Fig. 3) (Lim et al. 2022). The response of *gis-5* mutant plants to infection by Cabbage leaf curl virus is not known.

#### Watermelon

Variant data from 2 different, overlapping whole-genome resequencing projects revealed 2 *POLD1* missense mutations present in 8 watermelon accessions (*Citrullus* species) (Guo et al. 2019; Wu et al. 2023). These were predicted to have deleterious effects, though they were located outside of the domains affected in known geminivirus resistance-conferring alleles (Fig. 2b). Variants resulting in M228I substitutions were found in accessions WM98 and WM101 and in R371C substitutions in accessions WM98, WM99, WM101, WM102, WM426, WM427, and WM464 (Guo et al. 2019). The response to geminivirus infection in these accessions remains to be determined.

#### Cucumber

Analysis of a core set of SNPs in cucumber (*C. sativus*), derived from Genotype by Sequencing data for 1,234 accessions, revealed 2 missense mutations in *CsPOLD1* (CsGy6G025560). Neither is predicted to negatively affect protein function, nor are they found in the domains of interest in the POLD1 protein (Table 2).

#### Pigeon pea and *Me. truncatula*

Analysis of a first generation HapMap of 20 pigeon pea (*Cajanus*) species revealed 4 missense mutations in *CcPOLD1* (C.cajan_04111) (Kumar et al. 2016), one of which was predicted to negatively impact protein function (Table 2). The R370C mutation, identified in the wild species *Caj. acutifolius,* was located within the sequence encoding the exonuclease domain of POLD1 (Fig. 2b) and outside of the domains of interest.

Forty-four missense mutations were identified in *POLD1* from genome resequencing of 226 accessions of *Me. truncatula* (Stanton-Geddes et al. 2013), one of which was predicted to have a negative impact on protein function (G257A) (Table 2). This residue was not within the domains of interest (Fig. 2b). To date, a geminivirus pathogen has not been identified for either *Medicago* or *Cajanus* species, although many members of the *Fabaceae* family are hosts to geminiviruses (Jha et al. 2023).

#### Maize

Variant data from a diverse set of 1,498 maize varieties have been amalgamated and made available at the Maize Genetics and Genomics Database (Woodhouse et al. 2021; Andorf et al. 2025). Within the *Zea mays POLD1* gene (Zm00001eb201340), 14 missense mutations were present in the MaizeGDB 2024 High Coverage dataset (Table 2). Two mutations were predicted to negatively impact protein function, M529T and D896Y and were found in accessions CIMMYT landrace CHIS587 and Chinese landrace YASS-46, respectively (Wang et al. 2022), with the former SNP found in a region of interest (Fig. 2b). Response to infection by maize geminiviruses has not been reported for either accession.

#### Wheat

Targeted resequencing as part of the 1,000 exomes project generated variant data for 890 diverse wheat landraces and cultivars (He et al. 2019). As a hexaploid, the wheat genome is comprised of 3 subgenomes, where each encodes a copy of *POLD1*, TraesCS4A01G220100, TraesCS4B02G124700, and TraesCS4D01G094700. Analysis of each coding sequence revealed 8, 0, and 14 missense mutations, respectively (Table 2). Further analysis determined that 6 mutations were predicted to negatively alter protein function, with one mutation in the chromosome 4A copy (L562M) and the remaining 5 in the chromosome 4D copy. Mutation L562M was found within the region of interest (Fig. 2b); the response to infection by wheat geminiviruses remains to be determined in accessions with this mutation.

## Discussion

A total of 7 SNPs in *POLD1* were previously identified in geminivirus-resistant cassava and tomato as the underlying source of resistance. Here, the analysis of publicly available data and targeted cDNA sequencing has identified 5 additional SNPs in *POLD1* of geminivirus-resistant plants. Additionally, at least 9 new polymorphisms have been identified from resequencing projects as candidate geminivirus resistance alleles. These mutations cluster within the catalytic center of the palm motif, the finger motif, and one alpha helix of the N-terminal domain.

More than one mutated residue was observed in multiple species. One example was residue Y520 in cassava (Table 2), which corresponds to the Y515H mutation in the *Ty-6* locus of geminivirus-resistant tomato and Y514S in geminivirus-resistant soybean PI 171443. Y515H was found in the homozygous state in tomato, and the *Ty-6* locus had previously been described as conferring incomplete dominance to TYLCV where heterozygous individuals have an intermediate resistance response (Gill et al. 2019). Y514S in soybean PI 171443 was also found in the homozygous state, and geminivirus resistance has been described as a monogenic and recessive trait in accession SL525, the progeny of a cross between PI 171443 and a susceptible parent (Rani et al. 2017). Soybean has 2 genomic copies of GmPOLD1, one on chromosome Gm04 and the other on Gm06, making the homozygous mutation Y514S effectively heterozygous within the cell as both alleles of the copy on Gm04 encode Y514. Additionally, this same residue in cassava, previously identified as a site for a deleterious allele in the cassava haplotype map (Ramu et al. 2017) (Fig. 2b), was present in the heterozygous state in 4 cassava accessions as Y520S but was excluded from the list of polymorphisms linked to resistance to CMD as one variety had an average severity score of 2.67, indicating partial susceptibility (Lim et al. 2022). These data may suggest that a heterozygous mutation at this residue confers partial or incomplete resistance while robust resistance is observed when this mutation is in the homozygous state.

Another POLD1 residue affected by variation in multiple species was V528L in cassava, V534L in cotton, and V523I in tomato (Table 2; Fig. 2). The V523I mutation identified in *S. lycopersicum* LA1479 was in the homozygous state, and the response to geminivirus infection is not known for this variety. Cassava variety TME204, which contains a heterozygous V528L mutation, was asymptomatic when exposed to African cassava mosaic virus (ACMV) and displays a recovery phenotype when infected by the more virulent East ACMV (EACMV-K201), becoming asymptomatic by 65 d post inoculation (Kuria et al. 2017). Assessment of CLCuD resistance in glasshouse conditions determined that cotton accession Mac7 remained asymptomatic during the entire experiment, while the susceptible variety Karishma showed CLCuD symptoms as early as 14 d postgermination (Zaidi et al. 2020). The *G. hirsutum* genome encodes 2 copies of *POLD1*, and analysis of the available data indicated that the V534L mutation was heterozygous in that 2 alleles carry the mutation and 2 alleles do not. Unlike the Y520S and Y530H mutations discussed above, a mutation affecting this residue in the heterozygous state appears to be sufficient for resistance.

The residue identified in the temperature-sensitive *Arabidopsis* mutant *gis-5* as A707V was also found in geminivirus-resistant cassava as A684G. Arabidopsis plants with this homozygous mutation have early flowering and curly leaf phenotypes when grown at 28 °C but are unaffected when grown at 18 °C (Iglesias et al. 2015). Cassava plants with a heterozygous A684G mutation have no reported growth or development phenotypes. We predict that *gis-5* will display a robust resistance response to cabbage leaf curl virus and that resistance may be temperature sensitive.

Another residue of interest was identified in resistant *Cu. moschata* as a homozygous A671V mutation. Cassava variety NR87184 is heterozygous for a A676T mutation at this same residue (Ramu et al. 2017) but has a recorded average CMD score of 2.5, indicating at least partial susceptibility. Virus resistance in 2 resistant lines of *Cu. moschata* is described as recessive, and at least for line PVR-1343, multiple generations of phenotyping determined that resistance is controlled by 2 recessive genes (Verma et al. 2023a, 2023b). Indeed a second, smaller effect QTL was identified on chromosome 17 in PVR-1343 (Verma et al. 2023b), although the identity of the underlying gene contributing to resistance remains to be determined. Thus, we propose that the homozygous A671V mutation in *CmPOLD1* in variety PVR-1343 is likely the source of recessive geminivirus resistance at the chromosome 7 QTL and suggest that a homozygous mutant at this locus in cassava may also confer robust resistance to cassava mosaic virus.

Mutations were also identified in both maize and wheat within the N-terminal domain alongside known resistance alleles. Resistance to maize streak virus is an important trait to breeders and multiple QTLs have been identified as contributing to resistance, with the single locus *Msv1* on chromosome 1 a common source of resistance (Emeraghi et al. 2021). Identifying germplasm with putative resistance alleles in *ZmPOLD1* may be an additional source for introduction into breeding programs.

Resistance to geminiviral infection is a complex response. Indeed, in many species such as cassava (Okogbenin et al. 2012), peppers (Carrillo-Tripp et al. 2007), and cucurbits (Hagen et al. 2008), it often presents as a recovery phenotype where an infected plant initially shows symptoms, but new growth is less symptomatic and eventually virus-free, indicating a dynamic and multilayered defense response to the virus. The role of variant POLD1 proteins in resistance is still to be determined. One possibility is that polymerase activity is negatively impacted, leading to either an increase in errors during replication and/or slower replication efficiency of the geminivirus genome. In yeast, similar mutations have decreased accuracy and decreased polymerase activity (Daee et al. 2010). Deficiencies during virus replication may sufficiently alter the rate of virus accumulation and therefore provide time for antiviral defense responses. Better understanding of how mutations in *POLD1* lead to geminivirus resistance will also help to explain why some resistance requires homozygous variant alleles and other resistance is afforded by heterozygous alleles. Relatedly, certain deleterious mutations cannot be tolerated in a haploid or homozygous diploid state in yeast, presumably due to the essential role of POLD1 in eukaryotic cells (Daee et al. 2010). Further experiments will be required to determine how best to deploy high resistance-conferring alleles with no or minimal effects on growth and development.

### Conclusions

The analyses presented here suggest that *POLD1* variant alleles confer geminivirus resistance to a broad range of crop species, including cotton, soybean, and squash in addition to tomato and cassava. Specifically, we have identified putative causal mutations in *POLD1* of geminivirus-resistant varieties *G. hirsutum* Mac7, *G. hirsutum* NN-3, *Gl. max* PI 171443, and *Cu. moschata* PVR-1343. Additionally, putative resistance alleles were identified from resequencing data in rice bean, *Arabidopsis*, maize, and wheat. Together these data provide support that mutations in *POLD1* underlie geminivirus resistance in diverse species and that new sources of geminivirus resistance are likely available in existing germplasm collections.

## Supplementary Material

jkaf216_Supplementary_Data

## Data Availability

All data and code used in this work are available at FigShare DOI: https://doi.org/10.6084/m9.figshare.28585076.
